# The Hospitals Association

**Published:** 1891-10-31

**Authors:** 

**Affiliations:** Chairman of Council. 140, Strand, London, W.C.


### EDITOR'S LETTER BOX.

[Our correspondents are reminded that prolixity of statement is a great bar to publication, and that brevity of style and conciseness of statement greatly facilitate early insertion.]

### THE HOSPITALS ASSOCIATION.

Sir,—Will you allow me to draw attention to the Bill promoted by this Association with a view to modifying the restrictions hitherto prevailing in connection with devises of land to charities, and which became law at the end of last session? Briefly, the new Act (Mortmain and Charitable Uses Act, 1891) makes every gift by will of every kind of property good, only requiring that land given should be sold within a fixed period, the proceeds to become the property of the charity. It further has the important effect of excluding from all restriction, as regards disposition in favour of charity every kind of property to which the restriction formerly extended, except actual land. The cases in which land might be validly acquired by a charity under the old law are not affected by the new Act, the provisions of which, while removing the vexatious hindrances previously existing, in no way facilitate the permanent acquisition of land by a charity. Thanking you in anticipation, I am, sir, your obedient servant,

Carmarthen, Chairman of Council.

140, Strand, London, W.C., October, 1891.

